# ICH S1 prospective evaluation study and weight of evidence assessments: commentary from industry representatives

**DOI:** 10.3389/ftox.2024.1377990

**Published:** 2024-05-23

**Authors:** John L. Vahle, Joe Dybowski, Michael Graziano, Shigeru Hisada, Jose Lebron, Thomas Nolte, Ronald Steigerwalt, Kenjiro Tsubota, Frank D. Sistare

**Affiliations:** ^1^ Lilly Research Laboratories, Indianapolis, IN, United States; ^2^ Alnylam Pharmaceuticals, Cambridge, MA, United States; ^3^ Organon, Jersey City, NJ, United States; ^4^ Formerly ASKA Pharmaceutical Co., Ltd., Fujisawa-shi, Kanagawa, Japan; ^5^ Merck & Co., Inc., Rahway, NJ, United States; ^6^ Development NCE, Boehringer Ingelheim Pharma GmbH & Co. KG, Biberach an der Riss, Germany; ^7^ Takeda Development Center Americas, Inc., San Diego, CA, United States; ^8^ Astellas Pharma Inc., Ibaraki, Japan

**Keywords:** carcinogenicity testing, rat carcinogenicity, rasH2-Tg mouse dose selection, regulatory toxicology, carcinogenicity weight-of-evidence criteria, best practice

## Abstract

Industry representatives on the ICH S1B(R1) Expert Working Group (EWG) worked closely with colleagues from the Drug Regulatory Authorities to develop an addendum to the ICH S1B guideline on carcinogenicity studies that allows for a weight-of-evidence (WoE) carcinogenicity assessment in some cases, rather than conducting a 2-year rat carcinogenicity study. A subgroup of the EWG composed of regulators have published in this issue a detailed analysis of the Prospective Evaluation Study (PES) conducted under the auspices of the ICH S1B(R1) EWG. Based on the experience gained through the Prospective Evaluation Study (PES) process, industry members of the EWG have prepared the following commentary to aid sponsors in assessing the standard WoE factors, considering how novel investigative approaches may be used to support a WoE assessment, and preparing appropriate documentation of the WoE assessment for presentation to regulatory authorities. The commentary also reviews some of the implementation challenges sponsors must consider in developing a carcinogenicity assessment strategy. Finally, case examples drawn from previously marketed products are provided as a supplement to this commentary to provide additional examples of how WoE criteria may be applied. The information and opinions expressed in this commentary are aimed at increasing the quality of WoE assessments to ensure the successful implementation of this approach.

## 1 Introduction

The paper of Bourcier et al. (2024) entitled “ICH S1 Prospective Evaluation Study: weight of evidence approach to predict outcome and value of 2-year rat carcinogenicity studies. A report from the Regulatory Authorities subgroup” that appears in this issue of *Frontiers in Toxicology,* provides a detailed analysis of the Prospective Evaluation Study (PES) conducted under the auspices of the ICH S1B(R1) Expert Working Group (EWG). As described in the paper of Bourcier et al. (2024), these data supported the first change in the carcinogenicity assessment of small molecule pharmaceuticals since the introduction of alternative short-term mouse models in 1997. In fact, the ICH S1B(R1) Addendum (2022) could be viewed as the most notable change in carcinogenicity assessments for small molecule therapeutics since 2-year rat bioassays were first developed by the National Cancer Institute in the United States in the 1960s. The key element of the addendum is the provision of an option to conduct a weight-of-evidence (WoE) assessment of human carcinogenic risk in certain cases rather than conducting a standard 2-year rat study. While the addendum also describes a plasma exposure ratio-based approach for setting the high dose in the rasH2-Tg mouse model, the focus of this commentary is on the WoE option. For additional perspective related to dose selection for the rasH2-Tg mouse model refer to the analysis by Hisada et al. (2022).

This commentary was developed by industry members of the ICH S1B(R1) EWG and is meant to complement the information provided in the addendum to the ICH S1B(R1) guideline (2022) as well as the detailed review of the PES data by the regulatory authorities’ subgroup (Bourcier et al., 2024). Toxicologists and pathologists from industry were instrumental in the origins of what evolved into the WoE option starting with the work of scientists from industry in 2010 (Reddy et al., 2010) and then a seminal paper from a collaboration of scientists from Pharmaceutical Research and Manufacturers of America (PhRMA) member companies (Sistare et al., 2011). In addition, experience using a WoE approach was accrued for biotechnology-derived pharmaceuticals following the ICH S6(R1) Addendum (2011) and this experience helped inform the ICH S1B(R1) Addendum (2022). For a review of the origins of the WoE approach, please refer to the paper of Bourcier et al. (2024).

This commentary has several objectives. First, the paper will summarize best practice principles for sponsors and regulators to consider in determining when a WoE approach is appropriate. The standard WoE factors to consider are reviewed and then the role of investigative and emerging technologies is discussed since during the PES, data gaps or the need for clarifying information emerged as a key factor of discordance either between assessments by sponsors and health authorities or among the health authority reviews. Second, the paper will provide suggestions for sponsors regarding the documentation and presentation of WoE assessments to Drug Regulatory Authorities (DRA). As expected, during the PES, variability in the quality and format of documentation was noted by regulatory members and the goal of this section is to improve the quality of WoE documents submitted to DRAs. Third, the paper will discuss challenges for sponsors in implementing a WoE approach, which are important as these logistical and procedural challenges could threaten full utilization of the new approach. Lastly, the paper will provide case examples to illustrate the principles described in the revised S1B(R1) guideline (2022). These case examples are meant to complement and extend the case studies provided in the appendix to the ICH S1B(R1) Addendum (2022). While the content of this paper was developed based on the industry EWG members’ learnings and experience, to ensure industry input, this paper was reviewed by nonclinical groups within our constituent organizations (PhRMA, EFPIA, JPMA, BIO).

The ICH S1B(R1) EWG consisted of a highly engaged set of regulatory and industry members in an effort that spanned more than a decade. The authors of this paper appreciate the collaborative approach adopted by regulatory members of the EWG and acknowledge the sponsors who took the time and effort to contribute assessments and data during the PES without the possibility of direct benefit from the effort during the assessment period. We would like to thank the subset of regulatory members of EWG who analyzed and now published the key conclusions of the PES. While quite novel in the context of ICH work, the adjudication and analysis by this group of health authority scientists was essential to the success of the project.

The revision of ICH S1B (2022) to allow for a WoE option for carcinogenicity assessments in certain cases is a landmark change in carcinogenicity testing of small molecule pharmaceuticals; however, sponsors and regulatory assessors will need to effectively implement the guidance to maximize its full potential. It is important for sponsors to recognize the advantages of the WoE approach provides in assessing the safety of small molecule therapeutics. One advantage is moving from a “check the box” approach of conducting 2-year rat carcinogenicity studies to a more scientifically based approach that considers key pharmacologic and toxicologic properties for the compound. Another advantage is the opportunities for the expanded use of existing and emerging technologies to conduct more mechanism-based assessments related to assessing human carcinogenic risk. As outlined in the addendum, sponsors should rigorously assess the six primary WoE factors for all programs, not just those they consider suitable candidates for a WoE assessment. The rationale for doing the WoE assessment, even in cases where it does not result in elimination of the study, is to allow sponsors to probe potential gaps in knowledge or understand the molecules’ risks prior to testing. Importantly, in those cases in which the sponsor determines a 2-year rat study is warranted, they do not need to seek input from health authorities. In those cases, where a WoE determines a 2-year rat study is not warranted, we anticipate that this will avoid some of the inherent challenges of 2-year rat carcinogenicity studies such as equivocal outcomes or a positive finding which is later shown to lack human relevance. Finally, the WoE approach provides for a substantial reduction in animal use, as the standard 2-year rat carcinogenicity studies require between 500–700 rats.

It can be expected that in the early years of implementation, both industry and regulatory scientists may be cautious in adopting a WoE approach; however, we would anticipate that as sponsors and regulators gain more experience there will be increased opportunities to utilize a WoE assessment. Our hope is that the publication of Bourcier et al. (2024) which provides details from the data gathered during the PES, as well as this commentary sharing key learnings from industry participants in the ICH process, will increase the scientific rigor and effectiveness of future WoE assessments.

## 2 WoE factors

a) Like other ICH guidelines, the ICH S1B(R1) Addendum is not highly detailed in terms of the data or analysis the sponsor is expected to generate for each of the WoE factors. This is due to the fact that development programs will vary significantly based on the nature of the target or findings from the toxicology studies. In addition to program-specific considerations, sponsors vary in their strategy of certain aspects such as the scope of the secondary pharmacology screening data. For each of the factors listed, sponsors need to ensure they have generated a robust data set that allows them to decide on their carcinogenicity strategy and also provide regulatory scientists the data necessary to assess if a WoE approach is appropriate.

a) Target Biology

The pharmacologic activity and potency of the parent compound and major circulating metabolites should be considered for humans and in the animal species used for chronic toxicity testing. For further information on the definition of major metabolites refer to the ICH M3(R2) Guideline (2009) and its corresponding Question and Answer Document. This is frequently done by *in vitro* binding and activity assays using a recombinant cell line stably expressing the target and using inhibitory concentration (IC) or effective concentration (EC) values as an endpoint. The *in vivo* to *in vitro* ratio of these readouts serve to guide exposure targets and understanding concentrations necessary to reach full pharmacological efficacy. An additional target engagement parameter may substantiate the relevance of the *in vivo* model.

Drug target tissue distribution and pharmacological signaling cascades should be carefully assessed in rats and humans with a focus on actions relevant to carcinogenicity. This is generally done by use of open access and proprietary databases with example sources noted in Table 1, and complemented by review of relevant primary literature, which may also be referenced in databases. The general process of target safety assessment has been comprehensively described (Brennan, 2017). In addition, van der Laan et al. (2016) presented the outcome of their analysis of 298 pharmacological compounds with respect to their carcinogenic response per pharmacological class. This represents a valuable source of target-related carcinogenic risk for those established classes (van der Laan et al., 2016). Understanding interspecies differences in target distribution and pharmacologic pathways are of major relevance with respect to rat-to-human translatability. It is also important to review either publicly available or internal data on 2-year rat studies or other rodent carcinogenicity assessments conducted with other compounds in the class or compounds that have similar pharmacological properties. If such class-related carcinogenicity data are available, sponsors must consider how similar those compounds are to the molecule being developed in terms of potency, selectivity, pharmacokinetics, and toxicity profile.

**TABLE 1:** Examples of Information sources^a^ used in assessing target-related carcinogenic risk of small molecules.

Category	Database examples	Characteristics of data source
General characteristics of target protein and related gene	www.ncbi.nlm.nih.gov/gene	Public database of National Center for Biotechnology Information
Gene function	www.geneontology.org	Public database of GO consortium
Target distribution (rat, human)	https://www.proteinatlas.org/	Public database of the Swedish Human protein atlas (Uhlén et al., 2015)
	https://gtexportal.org/home/Genotype-Tissue Expression (GTEx)	Public database of the Broad institute
	www.biogps.org	Public database of The Scripps Research Institute
	https://www.frontiersin.org/articles/10.3389/fgene.2022.1078050/full	Gene expression tissue atlas published by Abbvie scientists of nonclinical tox species - rat, mouse, dog, NHP
Signal transduction/pharmacologic pathway downstream cascade	www.reactome.org	Public database of the Reactome Team (Ontario Institute for Cancer Research, European Bioinformatics Institute, New York University Medical Center)
	Ingenuity	Commercial database of Qiagen
Genetically engineered models	www.informatics.jax.org	Public database of The Jackson Laboratory/Mouse Genome Informatics (MGI)
Human genetic association studies	https://omim.org/	Public database of the Johns Hopkins University
	https://www.disgenet.org/	Public database of the Integrative Biomedical Informatics Group
	Open Targets https://genetics.opentargets.org/	Compendium of GWAS and WES rare variant associations
Cancer gene databases	https://portal.gdc.cancer.gov/	Public database of the Cancer Genome Atlas Program (TCGA) by the National Cancer Institute
	https://cancer.sanger.ac.uk/cosmic	Catalogue of Somatic Mutations in Cancer (COSMIC) by the Wellcome Sanger Institute
	https://www.intogen.org/search	Cancer Driver Genes Mutation Browser by IntOGen
Drug Approval Information	https://www.elsevier.com/products/pharmapendium	Pharmapendium provides publicly available information on marketed pharmaceutical

^a^ See also (Carss et al., 2023) “Using human genetics to improve safety assessment of therapeutics,” Nat Rev Drug Discov 22:145–162.

Important information sources also include phenotypic characterizations of genetically engineered animal models, human genetic association studies and cancer gene databases. Genetic variants that increase or decrease protein expression or function can inform on potential liabilities of agonistic or antagonistic target engagement, respectively. The relevance of any genetic association to pharmacological perturbation must consider multiple factors such as functional directionality, causality, penetrance, magnitude of effect, tissue distribution, etc. For a review including a listing of human genomics resources supporting human safety assessment see the manuscript by Carss et al. (2023).

Literature with relevance to target-related carcinogenic risk should be comprehensively searched in an unbiased manner, and documented as it serves as a key scientific building block to a thorough WoE assessment. Contradictory data should be mentioned with relevant context provided, as appropriate. Not all information on target biology has the same relevance for carcinogenic risk assessment, and it is important to provide an integrated analysis based on the totality of the data. Data from genetically modified animals, e.g., strains with a deleted or over-expressed target are generally considered to be of higher value than data generated *in vitro*, e.g., proliferation in a cell-based model. It is important to appreciate that homozygous gene deletion models may result in a phenotype that is more extreme and perhaps less relevant than that which would occur through pharmacologic modulation of a pathway that only partially abrogates signaling. Additionally, while cancer gene databases leverage sophisticated statistical algorithms to distinguish causal gene mutations, so called “driver” gene mutations, from “passenger” gene mutations, thresholds will vary, and inconsistencies are seen among databases. As in all scientific assessments, it is important to assess the overall quality of the publications with respect to the rigor of the model, group size, and methods of analysis with greater emphasis placed on those observations that are reproducible.

b) Secondary Pharmacology

Activity of a drug candidate and major metabolites at a pharmacological target other than the intended one, referred to as secondary pharmacology, has the potential to result in an increased carcinogenic risk. Such properties are assessed, in part, by secondary pharmacology screens, which are an integral part of drug candidate profiling (Jenkinson et al., 2020; Scott et al., 2022). However, standardization and best practices for screening methodology and targets is lacking. It is common practice to start by profiling drug candidates in an off-target *in vitro* panel in the lead optimization phase. Such early panels usually employ a limited number of targets and focus on functional effects and target organ toxicity. A commonly used panel is the one described by Bowes et al. (2012) that comprises 24 G protein-coupled receptors, 8 ion channels, 7 enzymes and 3 transporters but only 2 nuclear receptors and no kinases. Also, a recent compilation of potential adverse effects related to agonistic or antagonistic effects to 70 pharmacological targets (Lynch et al., 2017) is of limited value with respect to carcinogenic risk assessment as it focuses on common targets in pharmaceutical research and development.

Second tier screenings, conducted in a later phase of development, often as a part of the data to support Phase I clinical studies, may be more comprehensive and include targets with known carcinogenic risk, in particular kinases and nuclear hormone receptors. Examples may include the estrogen receptor (Duijndam et al., 2021), Glycogen Synthase Kinase 3 beta receptor (Heinemann et al., 2022) or the aryl hydrocarbon receptor (Podtelezhnikov et al., 2020). Under the auspices of the DruSafe leadership group of the Innovation and Quality (IQ) consortium there is ongoing work to comprehensively review current practices. Additionally, once major circulating metabolites are identified in the human ADME study, these metabolites should also be evaluated in secondary pharmacology screens.

As described above, secondary pharmacology screening strategies vary between sponsors. In fact, insufficient information on target selectivity arose as a deficiency in WoE assessment in several of the cases in the PES. As such it is important for sponsors in their WoE documentation to precisely describe the secondary pharmacology panels that were assessed and how those findings relate to carcinogenic risk. An emerging area is the inclusion of assays in off-target screening panels that specifically address the needs for a carcinogenic risk assessment. This is an area that will require additional investigation and input from the broader scientific community.

c) Histopathology

The guideline specifically emphasizes the importance of the 6-month chronic toxicity study in rats since data derived from these studies was foundational for the WoE concept. While the primary focus is on histopathology findings in rat chronic toxicity studies, results from repeat-dose toxicity studies in other species may also be helpful to assess the human relevance of a finding present in rats. For example, a finding that occurs in both rats and a nonrodent species is more likely to be of human relevance than a finding that only occurs in rats. Conversely, a finding of concern that occurred in the nonrodent only may warrant additional characterization but does not necessarily increase the need for a 2-year rat study, particularly if there are data such as species differences in potency or receptor distribution which indicate the rat is insensitive to the effect.

The ICH S1B(R1) Addendum (2022) specifies histopathology observations that are most often a risk factor including cellular hypertrophy, cellular hyperplasia, persistent tissue injury, chronic inflammation, foci of cellular alteration, preneoplastic changes and tumors. Each of these findings should be carefully considered, including their nature and magnitude. It is also important to note that some histologic findings may not have been considered adverse in the context of the repeat-dose toxicity study, but still need to be carefully considered in the WoE assessment. A low number of tumors are occasionally seen in 6-month rat studies and in many instances are spontaneous and unrelated to the test article (Son et al., 2010; Blankenship and Skaggs, 2013). For those instances where the occurrence of a tumor in a test-item treated group was considered spontaneous and this conclusion was well supported by historical control data, the data should be clearly described in the WoE documentation; however, these spontaneous tumors should not increase the need for a 2-year rat study. There are some cases in which the incidence of tumors in the 6-month study was considered of equivocal relationship to treatment. In these cases, such tumors must be considered in the overall WoE assessment, in particular if they can plausibly be related to other test-item related pathology findings in that tissue. To better understand the potential for proliferative findings to be preneoplastic, it is recommended to refer to standard texts of toxicologic pathology, in particular the standardized toxicologic pathology nomenclature documents and publications (goRENI)^1^.

The histopathologic risk factors should be considered in conjunction with any associated organ weight change. Organ weights can be a surrogate marker for hypertrophy or hyperplasia and cell proliferation if the cell compartment affected represents a major constituent of the respective organ, like hepatocytes in the liver, and there is no viable alternative explanation. On the other hand, a constant organ weight may not exclude increased proliferation: an increased cell loss by apoptosis may be counterbalanced by increased proliferation. For additional guidance on approaches to organ weight collection and interpretation refer to reviews and best practice recommendations by the Society of Toxicologic Pathology (Michael et al., 2007; Sellers et al., 2007).

For each of the histopathologic risk factors there may be cases where further investigation is warranted to assess biologic significance. For example, in some cases it may be warranted to quantitate cell proliferation or other associated parameters to aid in further understanding the nature of the finding. For further consideration on the utility of cell proliferation assessment in the context of the WoE assessment see Section 3.3.

d) Hormonal effects

Hormonal perturbation is known to represent a risk factor for nongenotoxic carcinogenesis in rodents and humans. The predominant mechanism of hormonal carcinogenesis is a sustained increase in cell proliferation induced by trophic hormones (Silva Lima and Van der Laan, 2000). The ICH S1B(R1) Addendum is comprehensive with respect to the parameters that may indicate a hormonal effect and it is important to highlight that the addendum is not suggesting that hormone levels be determined in the repeat-dose toxicity studies in rats. Changes in hormone levels are often difficult to assess in routine studies due to the interindividual variability, circadian rhythms, and analytical challenges (Stanislaus et al., 2012). Thus, even if hormone levels were evaluated in a study it may result in either a “false negative” or “false positive” with respect to an effect on circulating hormones. For evaluating potential hormonal effects, histopathology (hypertrophy and hyperplasia) and organ weights can be more robust endpoints. In cases of diffuse hypertrophy or hyperplasia, organ weight may be a more sensitive endpoint than histopathology, especially in cases of accessory male sex glands, adrenal, or pituitary. It is acknowledged that in the context of a 6-month rat study where reproductive senescence is occurring in some strains, it is important not to overinterpret a reproductive organ weight change that is not supported by corroborative findings. Sponsors should ensure that hormone-responsive organs are carefully collected and trimmed in chronic toxicity studies to minimize variability in organ weights due to tissue processing, especially if an effect on a hormonal axis is suspected.

Hormonal perturbation can be primary (e.g., direct interaction of the drug with a hormone receptor) or secondary (e.g., increased degradation of a hormone). With respect to secondary hormonal changes the addendum specifies that hormonal changes secondary to processes like stress or altered body weight are unlikely to be relevant to human risk assessment. In addition, in those cases where there has been sufficient mechanistic data that the hormonal effects in rats are a rodent-specific effect, a 2-year rat study would not be warranted based on this alone. Therefore, it is important that sponsors provide sufficient explanations of potential hormonal effects such as organ weight changes of endocrine or reproductive tissues to delineate primary vs. secondary effects. In some cases, this may warrant follow-up investigative studies that may include determination of circulating hormones on a case-by-case basis.

e) Genotoxicity

An absence of genotoxicity in a battery of tests conducted in accordance with ICH S2(R1) (2011) is an important component of a WoE assessment in concluding that a 2-year rat study is not warranted. ICH S2(R1) (2011) gives guidance on how to interpret positive or equivocal genotoxicity results from the standard test battery and suggests follow-up tests to de-risk these findings, including human relevance of the mode of action and the concentration threshold. Equivocal or positive data may require the identification of the mode of action of genotoxicity to identify if a molecule has intrinsic genotoxicity or not. For those programs where mechanistic approaches have not resolved uncertainty with respect to genotoxic potential, a 2-year rat carcinogenicity study would be warranted.

f) Immune Modulation

The addendum specifies that immune modulation, as characterized by the principles in ICH S8 (2005) on Immunotoxicity Studies, is an important WoE factor. While ICH S8 (2005) does not use the term immune modulation, it defines immunotoxicity in scope of the guideline as unintended immunosuppression or enhancement. Immunosuppression, however, is known to be associated with an increased tumor risk in animals and humans often due to reduced immune surveillance of tumorigenic viruses. Of particular note are B-cell lymphoma, squamous cell carcinoma and Kaposi sarcoma (Vial and Descotes, 2003). Building on the review of Bugelski et al. (2010), a workshop on cancer risk assessment of immunomodulators concluded that rodent carcinogenicity studies are generally not reliable predictors of human cancer risk associated with immunosuppression (Lebrec et al., 2016). Consequently, the ICH S1B(R1) Addendum (2022) states that human cancer risk assessment of a nonselective or particularly potent immunosuppressant will not be further informed by standard rat and mouse carcinogenicity studies. Examples of these types of agents include cyclosporine or tacrolimus. In such cases product labelling and post marketing surveillance will need to address the potential for increased risk for certain cancers wherein approval is otherwise warranted.

For programs where the pharmacologic intent is selective modulation of the immune system or there is an off-target effect that modulates some specific component of the immune system, sponsors should carefully assess the role the pathway plays in tumor immune surveillance to assess the potential risk along the principles outlined in the workshop report of Lebrec et al. (2016). The gradation of risk based on the specific immune pathway impacted can be illustrated in the product labelling of biotherapeutics that intend to modulate the immune system. Therapeutics that inhibit TNF (e.g., HUMIRA^®)^ carry bolded warnings for the risk of lymphoma and other malignancies based on human data (Food and Drug Administration US, 2002), while STELARA^®^ that binds to the p40 subunit of IL-12 and IL-23 carries a warning of potential risk of malignancy (Food and Drug Administration US, 2009), and COSENTYX^®^ which inhibits IL-17 does not carry a warning of increased malignancy risk (Food and Drug Administration US, 2015). Despite efforts by various laboratories over the years, there are no reliable broad screening models, either *in vitro* or *in vivo*, to reliably assess malignancy risk secondary to immune modulation. As such, the sponsor should assess if there may be more targeted, hypothesis-driven experiments that would inform risk related to the pathway that is being modulated. If there are no targeted experiments that might further inform risk the sponsor should provide an integrated analysis in their WoE documents and consider what types of product labelling and post marketing surveillance might be warranted. It is the view of the industry EWG members that in those cases where the only potential risk factor is immune modulation, a 2-year rat study is generally not warranted as it neither effectively identifies nor refutes a risk.

An additional challenge is for compounds that do not intend to modulate the immune system but have clear effects on one or few associated parameters of relevance. Such first evidence for immune modulation is often derived from repeat-dose toxicity studies and may include effects on white blood cell parameters, effects on immune globulins, changes in lymphoreticular organ weight, histopathology findings in lymphoreticular/hematopoietic organs, increased incidences of infections or increased occurrence of tumors in the absence of other plausible causes as summarized in ICH S8 (2005). Accumulation of a compound in lymphoreticular organs, derived from whole body autoradiography or histopathology/mass spectrometry, should also be considered when assessing the potential to impact the immune system. In these cases, the sponsor should consider if there are investigative approaches that could inform either human relevance or potential impact on immune surveillance. As discussed above, if the only potential risk factor identified are effects on the lymphoid system, a 2-year rat study would not be informative and thus, not warranted.

## 3 WoE factors–role of investigative studies and emerging technology

### 3.1 Nonclinical data to establish a strategy for assessment of human relevance

In addition to the six primary WoE factors discussed above, ICH S1B(R1) Addendum (2022) mentions non-standard end points or techniques that may further inform human carcinogenic risk assessment on an as needed basis. These investigations may be particularly valuable when there are findings of carcinogenic concerns in the *in vivo* studies. Such end points or techniques may be applied in additional investigative studies or to specimens collected from prior studies. Techniques that may be used more frequently will include special histochemical stains, immunohistochemistry, quantification of cell proliferation, molecular pathology, additional immunotoxicity studies according to ICH S8, and the various “Omics” technologies, among others. Since unexpected findings arise during the conduct of the repeat-dose toxicity studies, sponsors may choose to prospectively bank a subset of tissues, serum, or plasma in an appropriate manner from all of their repeat-dose toxicity studies to enable potential retrospective investigations. A drug-related finding should be characterized with appropriate additional techniques applied on samples from standard toxicity studies as early as possible in development. This will enable the inclusion of suitable non-standard end points in follow-up studies and help to reduce the need for stand-alone investigative studies.

The collaborative industry data mining publication by (Sistare et al., 2011) supporting the genesis of the ICH S1 revision proposal revealed that among all rat organs, the liver was the most common organ to have histopathologic risk factors of carcinogenicity in chronic toxicity studies. Furthermore, liver findings at 6 months were closely associated not only with eventual liver tumors but also with thyroid or testicular Leydig cell tumors. Biological explanations for the causal connections between a histopathologic risk factor in one tissue with tumors seen at alternate tissue sites have emerged over decades of rodent carcinogenicity testing. Efforts have been made to systemically catalogue these findings and this can be very useful in the early stage of a WoE evaluation for carcinogenesis assessment strategies.

During ICH S1 deliberations the EWG reviewed and acknowledged the value of such historical work underlying an expansive set of such multistep rat specific tumorigenic mechanisms collected over years of mining publicly available regulatory submission documents and published manuscripts by JPMA investigators and shared by JPMA representatives with the S1 EWG. The JPMA catalogued patterns of histopathologic risk factors of rat carcinogenicity observed among similar members of numerous pharmacologic classes and explained through investigative efforts to link chronic rat study findings to tumor types in a variety of endocrine and non-endocrine organs. This JPMA data survey has been presented publicly. This summary of historical perspective has been catalogued by JPMA using publicly available regulatory submissions on investigative successes applied in drug development to provide understanding of mechanisms, reduce human safety concerns, and support marketing authorization. Patterns of risk factors and associated tumors seen among 16 organs across common members of 28 classes of pharmacologic drug action are provided in Table 2.

**TABLE 2:** A summary historical perspective catalogued by JPMA from publicly available regulatory submissions on investigative successes applied in drug development to provide understanding of mechanisms of carcinogenesis, histopathologic risk factors (HPRF), and associated tumors seen among 16 organs across common members of 28 classes of pharmacologic drug action. This analysis, broken down into endocrine A) and non-endocrine B) mechanisms, was prepared as a resource for investigating tumorigenic mechanism when a positive result is obtained in a rat carcinogenicity study and/or for launching early investigations from patterns of HPRF in chronic studies and other available sources of pharmacologic and toxicologic information.

A. Endocrine tumors				
Drug–induced tumors	Drug class	MOA	HPRF	References for MOA
Pancreatic islet cell tumor	Serotonin-dopamine antagonists	Increased prolactin level	β cell hypertrophy/hyperplasia	Mortensen (1989), Brelje et al. (1994)
Thyroid follicular cell tumor	Hepatic enzyme inducers, Antithyroid, Iodide-containing agents	Increased TSH level	Thyroid follicular cell hypertrophy/hyperplasia	Hill et al. (1989), Thomas and Williams (1991), Hill et al., 1998; Hurley (1998)
Thyroid C cell tumor	GLP-1 agonists	Direct agonistic effects	Diffuse/focal thyroid C-cell hyperplasia	Bjerre Knudsen et al. (2010), Parks and Rosebraugh (2010), Hegedüs et al. (2011), Gier et al. (2012), Madsen et al. (2012)
Adrenal pheochromocytoma	Ca channel antagonists, Polyols, PDE3 inhibitors, Vitamin D3, Retinoids, SGLT2 inhibitors, a-glucosidase inhibitors	Sympathetic stimulation	Diffuse/nodular hyperplasia of adrenal medullary cells	Lynch et al. (1996), Tischler (1999), Greim et al. (2009)
Leydig cell tumor	Anti-androgens, 5a-reductase inhibitors, testosterone synthesis inhibitors, aromatase inhibitors, D2 agonists, PPARα agonists, polyols, a-glucosidase inhibitors, SGLT2 inhibitors, LH-RH agonists	Increased LH level	Leydig cell hyperplasia	Prentice et al. (1992), Clegg et al. (1997), Cook et al. (1999)
Mammary tumor	D2 antagonists, SDA, Estrogens, synthetic estrogens, progestogens	Increased prolactin level	Mammary gland hyperplasia (lobular, ductal)	Blum et al. (1987), Alison et al. (1994), Harvey, 2012; Vyas (2012)
Anterior Pituitary tumor	LH-RH agonists, D2 antagonists	Unknown, antagonism of inhibitory effects on proliferation	Hypertrophy/hyperplasia, anterior pituitary	Donaubauer et al. (1987), Saiardi et al. (1997), Heaney et al. (2002), Iaccarino et al. (2002), Hnasko et al. (2007), Greaves (2012a)
Endometrial tumor	Dopamine agonists	High estrogen/progesterone	Endometrial hyperplasia	Griffith (1977), Ben-Jonathan et al. (2008), Hargreaves and Harleman (2011), Greaves (2012b)

B. Non-endocrine tumors				
Drug –induced tumors	Drug class	MOA	HPRF	References for MOA
Hepatocellular tumor	Hepatic enzyme inducers, PPARα agonists, Synthetic estrogens	Activate target molecules/receptors: CAR, PPARα	Clonal expansion of preneoplastic foci	Kawamoto et al. (1999), Yamamoto et al. (2004), Holsapple et al. (2005), Corton et al. (2014)
Pancreatic acinar cell tumor	PPARα agonists, Trypsin inhibitors	Increased CCK levels	Pancreatic acinar cell proliferation	Douglas et al. (1989), Bourassa et al. (1999), Moore et al. (2001), Pandiri (2014)
Renal tubular tumor	α-glucosidase inhibitors, SGLT2 inhibitors, SERM	Ca imbalance by carbohydrate malabsorption	Regenerative hyperplasia of tubular cells	Hard (1998)
Hemangiosarcoma, subcutaneous sarcoma	PPARγ agonists, PPARα/γ agonists	Accelerate cell proliferation, unknown detailed mechanism	Increased epithelial proliferation in mice	Hardisty et al. (2007), Cohen et al. (2009), Criswell et al. (2012)
Urinary bladder tumor	PPARγ agonists, PPARα/γ agonists	Prolithogenic mechanism	Regenerative hyperplasia of bladder epithelium	Burin et al. (1995), Cohen and Lawson (1995), Cohen (1998), Hardisty et al. (2008)
Gastric carcinoid	Anti-secretory agents, e.g., H2-blockers and PPIs, PPAR α agonists	Hypergastrinemia	Gastric ECL cell hypertrophy/hyperplasia	Håkanson and Sundler (1990), Robinson (1999), Lamberts et al. (2001)
Hibernoma	Nicotine receptor agonist, α receptor blocker, opioid agonist, JAK inhibitor	Sustained sympathetic stimulation	Brown adipocyte hyperplasia	Cannon and Nedergaard (2004), Sell et al. (2004), Radi et al. (2013)
Mesovarian leiomyoma	β2-agonists	Direct agonistic effects	Mesovarian smooth muscle cell hyperplasia	Poynter et al. (1978), Gopinath and Gibson (1987), Kelly et al. (1993)

HPRF, histopathologic risk factor; LH-RH, Luteinizing Hormone-Releasing Hormone; MOA, mode of action; PDE, phosphodiesterase; SDA, serotonin dopamine antagonist; SGLT2, sodium glucose co-transporter 2.

CAR, constitutive androstane receptor; CCK, cholecystokinin; HPRF, histopathologic risk factor; JAK, janus kinase; MOA, mode of action; PPAR, Peroxisome proliferator-activated receptor; PPI, proton pump inhibitor; SERM, selective estrogen receptor modulator; SGLT2, sodium glucose co-transporter 2.

Under the Organisation for Economic Co-operation and Development (OECD) Adverse Outcome Pathways (AOP) Programme^1^, the OECD is systematically constructing a public knowledge base by collecting AOPs on the development of human and environmental hazards on its website, the AOP Wiki^2^, with the goal of developing a defined Integrated Approach to Testing and Assessment (IATA) for use in regulation, that is becoming a similarly valuable resource for sponsors. The AOP consists of a molecular initiating event (MIE), an adverse outcome (AO), and multiple key events (KEs) in a pathway from the MIE to the AO. Measurements for each KE are described in the KE sections and the scientific and quantitative plausibility of the relationship between KEs are described in the sections of Key Event Relationships (KERs). As of April 2023, there are 23 AOPs under development on human carcinogenesis and 25 AOPs on multi-step mechanisms of rodent-specific carcinogenesis summarizing and documenting research conducted over decades.

The JPMA data survey and the AOP WIKI are extremely valuable resources to sponsors who could access such prior precedent in seeking to meet the expectation of satisfactorily addressing tumorigenic risk potential of 6-month rat study findings in accordance with the S1B(R1) Addendum (2022). Nonclinical investigative methods applied to development programs that are based on historical documentation can be useful for guiding construction of explanations for those commonly observed patterns of histopathologic risk factors associated with frequently encountered on- or off-target mechanisms involving excessive and sustained pharmacology or toxicology. It is important to remember that these historical compendia began as individual customized project-specific approaches involving unique and creative nonclinical investigative strategies. While learnings from these historical examples can be informative, it is essential to have appropriate bridging study data to support any claims of applicability to new compounds in development.

### 3.2 Clinical data to assess human relevance

In a similar manner it may be informative to include translational biomarkers that can inform critical aspects of tumorigenic mechanism, or specific organ safety biomarkers in clinical studies to help obtain information on the human relevance of toxicities identified in rats, when available. As pointed out in ICH S1B(R1) (2022), such human clinical trial or epidemiologic data can also be useful by providing critical human perspective to novel mechanisms underlying potential risks raised by the WoE criteria or to address findings that cannot be readily accounted for by prior established mechanisms. These may often, but not always, involve engagement of intended on-target or closely related pharmacologic targets. The initial observation of osteosarcomas in rats seen with FORTEO^®^ first approved in the US in 2002 for treatment of osteoporosis and limited initially to use in post-menopausal women deemed at high risk for fracture, and for a limited duration of 18 months treatment, represents such an example of integrated nonclinical and clinical investigation summarized by Miller et al. (2021). In 1998 findings of osteosarcoma in the rat carcinogenicity study triggered a halt to ongoing clinical trials, and the sponsor Eli Lilly and Co., conducted long term studies in monkeys demonstrating the osteosarcoma risk to be mechanistically unique to rodents whose skeletal growth continues through life, while growth plates of primates will close (Vahle et al., 2002). While such data allowed for initial limited marketing approval, subsequent epidemiologic studies provided further confirmation of the lack of osteosarcoma risk to humans leading in 2020 to an improved benefit-risk appreciation with extension of labeled dosing duration, expansion of the indicated population, and relaxation of the carcinogenic label warnings (Krege et al., 2022).

An additional example of the need for pivotal clinical data to support marketing approval and regulatory decision making is omeprazole and other proton pump inhibitors that induce neoplasia of enterochromaffin-like cells in rats (Ekman et al., 1985; Olbe et al., 2003). These molecules indirectly lead to increased gastrin levels that, in the rat, cause hyperplasia and neoplasia of gastric enterochromaffin cells. Similar findings using clinical gastrin monitoring and endoscopic imaging are not seen in humans receiving chronic therapy with proton pump inhibitors (Massoomi et al., 1993).

It is interesting to note how the passage of time enabled accrual of pivotal clinical data allowing, in the case of Forteo, for relief of restrictive labeling, expansion of the patient population, and relaxation of rodent carcinogenicity study label warnings. And in the case of proton pump inhibitors this therapeutic class started with black box warnings for rat tumors and was eventually judged to be sufficiently safe to allow purchase without a prescription as an OTC product. The challenge for industry scientists is to be mechanistically proactive and to apply existing and emerging tools that help to resolve questions of carcinogenicity risk.

### 3.3 Quantification of cell proliferation

As earlier described there may be occasions when targeted investigations of cell proliferation should be considered. Increases in cell proliferation are caused either by a direct stimulus via hormonal or nuclear receptors or indirectly as a regenerative response to cell death. An increase of cell proliferation represents a key event in basically every nongenotoxic carcinogenic MoA or AOP; however, an increase in cell proliferation at a single time point does not always result in an increased tumor risk. Since tumors can originate from increased cell proliferation leading to incorporation of mutations providing cellular growth advantage, establishing the threshold dose for cell proliferation can provide a rationale for the dose-related prediction of a nongenotoxic based tumor outcome (Cohen and Ellwein, 1990) and an evidence-based assessment of the clinical relevance of increases in cell proliferation. The assessment of cell proliferation traditionally requires a dedicated study or at least dedicated investigations. Such investigations will not be conducted routinely but rather for a specific purpose, usually based on certain histopathology findings, organ weight changes, or from theoretical considerations. An increase in cell proliferation can only roughly be assessed morphologically by routine semiquantitative histopathology because of the short duration of mitosis in the cell cycle and the rarity of mitotic figures in histological slides. Regenerative cell proliferation may be indirectly assessed by evidence of sustained cell damage like single cell necrosis and associated inflammatory reactions and the morphologic appearance of some cell types (epithelial basophilia). At lower levels of injury, however, cell loss may be limited to apoptosis, which is much more difficult to assess by routine histopathology.

This indicates that assessment of cell proliferation may represent an important follow-up activity for findings in repeat-dose toxicity testing (Wood et al., 2015). Proliferation kinetics differ based on the underlying mode of action, tissue and chemical, and need to be taken into consideration when planning for their assessment (Wood et al., 2015). Cell proliferation can be assessed by a variety of methods, and it is likely advances in digital imaging and analysis may lead to improved, more efficient methods in the future. Examples of methods currently available for use include immunohistochemistry for Ki-67 on archival sample, artificial intelligence-assisted counting of mitotic figures (Heinemann et al., 2022) or in the context of a prospective investigative studies BrdU-labelling (Nolte et al., 2005; Wood et al., 2015). Once experimental variables are optimized and sufficient data are gathered to understand normal variability in the model, cellular proliferation can be a valuable early endpoint for exploring and establishing mechanistic understanding of tumor pathogenesis. Given the criticality of experimental timing to capture a significant proliferative signal, its routine use for establishing negative predictivity can be a challenge.

### 3.4 Emerging role for genomic and genetic approaches

The emergence of genomic and genetic tools for predicting carcinogenicity are additional factors that can be considered when generating a WoE approach for carcinogenic risk assessment. These approaches also raise interesting questions. For example, what is necessary and sufficient to associate a well-documented mode of action with a prior established AOP to readily explain findings of concern identified in a chronic rat study as being either human relevant or irrelevant? Can genomic signatures be qualified for such an application? A collaborative approach (Corton et al., 2022) has been launched within the Health and Environmental Sciences Institute (HESI) as a direct response to begin leveraging such opportunities created by ICH S1B(R1) (2022). The initial aspect of this collaboration seeks to develop and qualify biomarker gene expression signature panels focused initially on rat liver that measure widely accepted molecular pathways linked to commonly observed tumorigenic mechanisms. Growing evidence suggests that application of such biomarker panels in short-term exposure rodent studies can readily identify both tumorigenic hazard and tumorigenic activation levels for certain chemical-induced carcinogenicity mechanisms. Success from these efforts focusing initially on rat liver is expected to help facilitate the transition from the currently heavy reliance on conventional 2-year rodent carcinogenicity studies to more rapid animal- and resource-sparing and earlier approaches for mechanism-based carcinogenicity evaluation supporting internal and regulatory decision-making.

An additional component of the HESI collaboration seeks to apply error-corrected sequencing (ECS) to identify early clonal expansion of growth advantaged cells harboring cancer driver gene mutations. While good progress has been made in demonstrating the value of ECS for identifying and examining mutations in key cancer driver gene mutation hotspots as biomarkers of *in vivo* genotoxic risk (Parsons, 2018; Merrick, 2019; Valentine et al., 2020), utility for nongenotoxic chemical tumor risk is only beginning to be explored and will require thorough validation and qualification for both sensitivity and specificity before being broadly adopted in nonclinical safety assessment. In the future, approaches such as ECS may be particularly useful for programs with novel pharmacologic targets (i.e., first-in-class molecules) by providing additional assurance that there are no molecular patterns indicative of clonal expansion in key target tissues.

### 3.5 Consideration of *in silico* approaches in the weight-of-evidence

Computational approaches for identifying structural alerts underlying genetic toxicology and carcinogenic risk (Smith et al., 2016) have proven to be very valuable. Early on, just prior to the initiation of the ICH S1B revision process, a proposal from FDA chemists was made for applying *in silico* tools to the 200+ compounds used in the PhRMA analyses. An analysis was conducted by the FDA chemists and no convincing argument could be made for adding this element to the WoE for carcinogenicity (Personal communication, Frank Sistare). Since so many diverse mechanisms underly the range of tumors observed, this outcome is not surprising. While *in silico* applications to carcinogenicity hazard assessment are likely to evolve (Tice et al., 2021), *in silico* predictions of carcinogenicity beyond mechanisms involving certain genotoxic mechanisms, have not been broadly accepted by the industry or DRAs and so are viewed as not presently ready as a routinely deployed WoE tool for carcinogenicity risk assessment.

### 3.6 Perspective on role of investigative studies

As described in Section 3.1. and Section 3.2. above, investigative strategies have long played an important role in carcinogenicity risk assessment; however, these efforts largely focused on understanding the human relevance of a rodent tumor finding that arose in standard carcinogenicity tests. Under the WoE option there now emerges the potential to leverage investigative approaches and newer technology to prospectively address potential risks. During the PES, investigative approaches to characterize potential carcinogenic risks were not common and none of the emerging genomic or *in silico* approaches described above were included in the submissions. It is critical to point out that during the PES, sponsors were still required to conduct a 2-year rat study and therefore were likely less proactive in generating data to explain the mechanism or assess human relevance of any finding that suggested a potential carcinogenic risk. During the PES an incomplete explanation of findings from the 6-month rats study findings was a common reason for disagreement between DRAs and sponsors and in some cases among or within DRAs.

In the future, strategic use of both existing models and methods as well as emerging technology will hopefully expand to provide a more mechanistic approach to carcinogenicity risk assessment as well as increase the number of programs which can utilize a WoE assessment. Investigative approaches may be particularly important to meet the higher evidentiary standard for first-in-class molecules. As described in Section 3.4 above, ECS may emerge as a tool to support a WoE assessment for new targets. Sponsors should not forget the potential for existing models in this regard. For example, when a pharmacologic mechanism can be activated similarly in rats and mice, then one could argue that the absence of tumor findings in the 6-month rasH2-Tg mouse study are additional supportive evidence that on-target activation presents lower risk for carcinogenicity. The recent RORgT example exemplifies the value of the short-term rasH2-Tg mouse model for identifying such on-target risks of novel first-in-class therapeutics (Haggerty et al., 2021). To be clear, the guidance does not require that a rasH2-Tg mouse study be completed prior to seeking agreement on a WoE approach. The point here is that sponsors may want to consider the conduct of a rasH2-Tg study sufficiently early to support such on-target risk assessments.

## 4 Best practices for WOE documentation

Once a sponsor has done an integrated analysis of the WoE factors and determined that a 2-year rat study would not contribute to human risk assessment they must document their WoE assessment for review by the DRAs. As a reminder and as specified in the guidance, formal documentation, and submission to DRAs is not required in those cases where the sponsor chooses to perform a 2-year rat study. The following provides suggestions for sponsors to consider based on the authors experience during the ICH process.

Evidence sources linked to the WoE criteria for *in vivo* studies will be primarily from standard toxicology studies (e.g., the standard genetic toxicology battery, histology from subchronic and chronic rodent studies, reproductive toxicology studies, secondary pharmacology screens, etc.) to minimize the need for additional animal studies. As such, collection and documentation of data for building the WoE document can be started early in each program to enable an early decision on whether it is feasible to pursue a WoE approach and/or whether additional information that needs to be generated to support a gap in a WoE endpoint is within the constraints of the project resources and timeline (see Section 5, Implementation Challenges Section).

**FIGURE 1:**
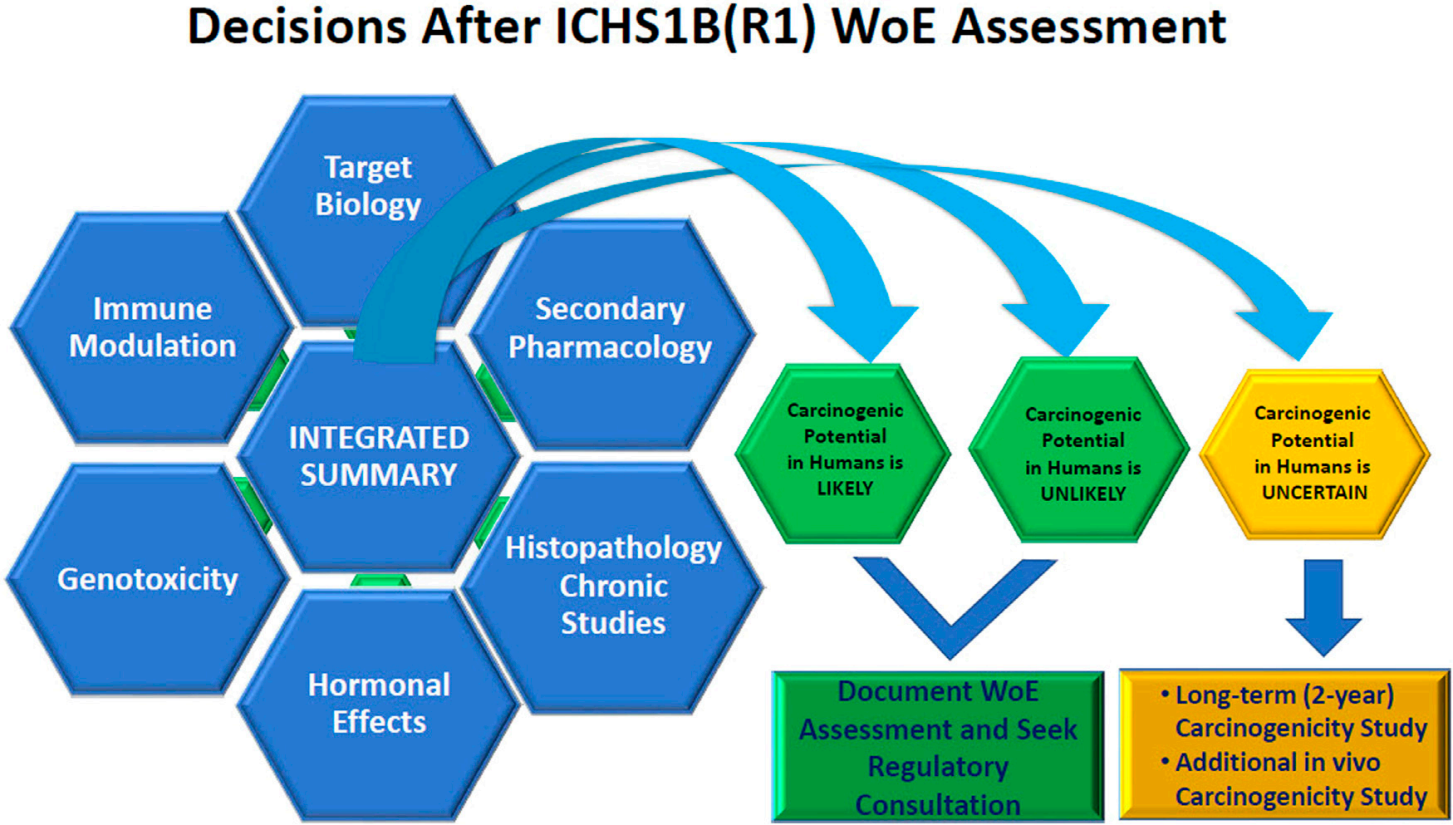
Visualization of the Integration of Weight of Evidence Factors to arrive at the conclusion of whether a 2-year rat study adds value to the human carcinogenicity risk assessment.

The summary of relevant information extracted from these studies in the WoE assessment should be focused as to how the key data from each study specifically relates to the carcinogenicity risk (e.g., what targets relevant to carcinogenicity risk were included in the *in vitro* secondary pharmacology screens to rule out secondary pharmacology as a carcinogenic risk or what clinical pathology and histology endpoints were evaluated to determine lack of immunotoxicity or hormonal effects in repeat dose toxicology studies). Figure 1 provides a pictorial overview of the process. The discussion should be balanced and indicate if gaps in data exist and the strength of the assessment of each factor in supporting the final WoE conclusion should be stated.

As part of the literature assessment, different lines of evidence can be explored, including publicly available carcinogenicity data on other chemicals within the same primary pharmacological class, the extent to which the biological pathways are well-characterized relative to potential involvement in cancer development and relevant carcinogenicity risks related to the pharmacology of any major human metabolites.

As with any regulatory submission sponsors should organize the information in a logical manner. Sponsors have the flexibility to organize the information to best suit the needs of the program. The following potential outline provides suggestions on key elements to include in their WoE submissions to DRAs.

a) Executive Summary
  • Provide a high-level yet integrated Executive Summary of the information gathered for each of the WoE factors.
  • Summarize the strategy taken to build the WoE including data sources and a discussion of which factors provide the strongest evidence to support the WoE overall conclusion with a balanced assessment of the factors that either have gaps in information or which do not clearly support the overall WoE assessment outcome.
  • Based on the overall balance of the WoE assessment, a clear assessment of whether the compound presents a high or low level of human carcinogenic risk, and how the data support the rationale for not performing the 2-year rat study should be provided. If necessary, justification for any alternative carcinogenicity assessment studies (e.g., “additional *in vivo* tests for carcinogenicity” as described in 4.2.2 of the ICH S1(R1) guidance (2022)) to complete the assessment should be discussed.
b) Materials and Methods
  • Either as a discrete section or embedded in the WoE Factor sections, sponsors should consider indicating which databases were used in their assessment and may want to elaborate on their literature search strategies.
  • An outline of *in vitro* and *in vivo* studies used to provide support for each of the WoE factors may be useful.
  • Outline any additional investigative studies or details of specific measurements taken during standard toxicology studies that were used to support the WoE conclusions.
  • Describe clinical sources of information, if applicable.
  • Hyperlinks to study reports and literature references can simplify the review process.
c) WoE Factor Subsections with Detailed Analysis
  • Each of the 6 WoE factors should have a dedicated section with a detailed discussion addressing the concepts from the ICH S1B(R1) guidance (2022). Refer to Section 2 of this commentary for specifics on each of these WoE factors. These sections should be primarily high level and strategically directed at discussing how the WoE factor contributes to the carcinogenic risk assessment with the bulk of experimental results referenced in appendices.
    o Target Biology
    o Secondary Pharmacology (including listing of targets screened)
    o Histopathology from chronic studies
    o Hormonal Effects
    o Genetic Toxicity
    o Immune Modulation
  • Carcinogenic risk of major human metabolites should be assessed and addressed if applicable.
  • Results of any investigative approaches as described in Section 3 of this commentary should be integrated into the appropriate WoE factor section.
  • Assessment of pharmacokinetics, and exposures in each study used for supporting each of the WoE factors (including parent and metabolites relative to secondary pharmacology, in addition to *in vivo* studies) with exposure multiples relative to maximum human exposure should be provided. A summary table including all the studies discussed will facilitate the interpretation.
d) Integrated Risk Assessment/Conclusions
  • The document should provide a conclusion section summarizing the WoE from each factor in support of the primary goal of determining whether a 2-year rat study would not add value to the human carcinogenicity risk assessment.
  • Sponsors might consider placement of each WoE factor on the “sliding scale” of Figure 2 of the ICH S1B(R1) guidance (2022) to help visualize the overall “weight” of each factor.
  • An overview of the proposed full carcinogenicity risk program (e.g., any additional investigative studies, additional *in vivo* carcinogenicity, etc.) should also be included.
  • While the ICH S1B(R1) Addendum (2022) does not specifically address clinical safety data, sponsors should assess the clinical safety data available to date and integrate into the overall risk assessment.

Note that in some cases the use of appendices may be useful to provide details on any aspect of the WoE assessment including nonclinical study summaries, tabular data, graphical data from databases, or other information.

## 5 Implementation challenges

To ensure the successful implementation of the ICH S1B(R1) Addendum (2022), it is vital for industry and regulatory scientists to maintain open communication and collaboration. Under this Addendum, sponsors have the burden of proof to make the case as to whether a 2-year rat carcinogenicity study would not add value to understand human cancer risk. As described above, the WoE assessment supporting the conclusion that a 2-year rat carcinogenicity study does not add value will need to have comprehensively addressed each of the WoE factors outlined in ICH S1B(R1) (2022), in addition to other relevant information, and present a rigorous, critical, and objective science-based assessment. In addition to the scientific considerations described previously in this paper, sponsors face important regulatory and logistical challenges that are summarized below.

### 5.1 Need for a predictable regulatory assessment process

Among the various implementation challenges to consider, likely the most important is the need to establish a predictable regulatory assessment process that is well defined, transparent, and dependable with reasonable timelines. The reason being that planning, execution, and finalization of a 2-year rat carcinogenicity study can take up to 4 years, as detailed below.

(1) Pharmaceutical companies work with Contract Research Organizations (CROs) or internal schedulers to schedule the study. Usually, this needs to be done at least 1 year in advance, as integrating these long-term studies into the test facility schedule can be challenging. Additionally, nonclinical safety organizations within pharmaceutical companies coordinate their study planning with clinical, formulation, chemistry, and all other functional areas within their organization so that carcinogenicity study completion will not be rate limiting for filing a marketing application.
(2) For US submissions, special protocol assessments (SPA) outlining the proposed study design, final draft protocol, and dose selection rationale are generally submitted for regulatory review and concurrence (i.e., FDA Executive Carcinogenicity Assessment Committee [eCAC] process) multiple months ahead of the anticipated carcinogenicity study start. The SPA process is unique to the FDA and similar processes are not in place in other regulatory regions. These activities occur in parallel to the study scheduling described in point 1) above with adequate time to adopt revised designs that may change scheduling study start and/or reporting timelines (e.g., changes in dose selection requiring securing additional drug, recommendations for alternative/additional controls requiring additional animal rooms, staffing resources and/or ensuring timely animal orders); importantly, these are amongst the aspects managed by organizations and/or with CROs to ensure there is no impact to filing a marketing application.
(3) Once the study is initiated, the in-life study activities will require 2 years.
(4) The post-mortem activities, including histopathological evaluation of a list of >40 tissues/animal for 500–700 animals, statistical evaluation, preparation of the study report, and QA review can take a year or longer.
(5) Finally, any additional evaluations/investigations to assess the risk associated with potential observations on a 2-year rat carcinogenicity study are to be factored in the overall timelines.

Additionally, it will be important for the sponsors to factor in the availability of the 6-month rat chronic toxicity data, as this is the most critical factor in the WoE assessment, and not typically conducted until later in the drug development timelines. This is especially important when the timeline of the Phase three clinical program is relatively short, as availability of chronic toxicity studies to first registration may not allow sufficient time for seeking regulatory feedback on the WoE assessment and conduct of a 2-year carcinogenicity study if one of the DRAs were to require it. However, a draft report of this study that includes the final audited integrated data (including a signed pathology report) should typically be sufficient for the DRA review of the WoE assessment. An additional key data set that sponsors also need to factor in when planning the preparation of the WoE assessment is the human metabolite data, as potential carcinogenic risks of major circulating metabolites must be considered.

Given the potential for multiple factors that may extend the timeline for planning and execution of a 2-year rat carcinogenicity study, it is imperative that WoE assessments be integrated in the overall drug development timelines. Therefore, concurrence or feedback from DRAs on the WoE assessment is needed well in advance of the marketing application filings, so that if a 2-year rat carcinogenicity study is needed, it can be executed in a timely manner, and not delay submission of marketing authorization applications and timely access of medicines to patients. In addition, it would be important to have a predictable review process. In discussions among the industry representatives who provided input on the implementation of the S1B(R1) Addendum (2022), it was suggested that a 3–4 months review period to complete the WoE assessment would facilitate efficient and timely drug development process.

### 5.2 Need to seek separate input from multiple regions

As drug development is an increasingly global process, registration is most often pursued in multiple regions in parallel. As such, requests for feedback would need to overlap for each of the agencies and it would only take one DRA to indicate that a 2-year rat carcinogenicity study is considered necessary for the sponsor to have to conduct the study. As ICH does not have as a part of their remit to provide a centralized source of regulatory review, sponsors need to seek feedback on the necessity for a 2-year rat carcinogenicity study from DRAs separately in countries where marketing approval will eventually be sought. Given the approximate 4-year timeframe for planning and completing 2-year rat carcinogenicity study-related activities as described above, it becomes critical for sponsors to determine the appropriate timing for submitting a WoE assessment based on the duration of the DRA review cycles.

A key question that would need to be answered is how many agencies to seek feedback from? The answer to this will depend on the registration strategy an individual sponsor takes, but generally companies seek first approval in the three major regions (US, Europe, and Japan), before seeking approval in other countries. Although discussions will occur between the company and each of the additional countries where the marketing application will be submitted, by then input from the three major regions will be known and can help companies get a sense on whether DRAs agree that conducting a 2-year rat carcinogenicity study would not add value to the human carcinogenicity risk assessment.

### 5.3 Intra- and inter-DRA discussions are encouraged as well as continued dialog with industry partners

As part of the regulatory review of a WoE assessment submission, it will be important that the feedback provided to sponsors reflects an aligned, actionable perspective from within the DRA. In this respect, we encourage DRAs to establish a central expert group within their organization to provide a final recommendation that ensures intra-agency alignment. This centralized expert group could also help coordinate input to the ICH S1B(R1) Implementation Working Group (IWG) being assembled to facilitate sharing experiences among DRAs on the outcome of the WoE assessments to help understand opportunities for improving the review process. Likewise, this IWG would allow industry members to receive feedback from DRAs on how best to improve the quality of submissions or sharing key learnings from the early stages of implementation.

The importance of such a process can be illustrated wherein the same drug and same dose are used in different patient populations, and where primary review of safety information may be undertaken by different regulatory scientists within the same agency, potentially at different points in time. As long as no substantial new scientific information relating to carcinogenic potential has become available, the central HA expert group could help maintain alignment. It would be troubling to have different conclusions on the value of the 2-year rat carcinogenicity study in contributing substantially to the human risk assessment for cancer for distinct but similar patient populations.

For transparency, individual sponsors may, as appropriate, play a proactive role in communicating to each of the DRAs when other DRAs have also received the WoE assessment submission, and if known, what the input received has been. As mentioned above, if at least one DRA asserts that a study is needed, then companies would need to trigger the conduct of the 2-year rat carcinogenicity study.

### 5.4 Labelling implications of not doing a 2-year rat carcinogenicity study

Currently results of rodent carcinogenicity studies are a standard part of product labeling for small molecule therapeutics. In many cases the labelling simply provides the results of those rodent studies and for those with positive rodent tumor findings often indicate that the relevance to humans is unknown. With the adoption of a WoE assessment option for some programs, it will provide regulators and sponsors an opportunity to reconsider how to make labelling of carcinogenic potential more useful for healthcare providers. The experience with labeling for biotherapeutics that have used a WoE assessment is highly variable and ranges from “carcinogenicity not assessed” to high level summaries of the WoE assessment. In the future, for programs which have used the WoE assessment option, the results of the rasH2 mouse study would likely be included in the labelling and it would be helpful if the high-level conclusion from the WoE assessment would also be included. For example, “An integrated analysis of available data suggested the potential carcinogenic risk of xxx is low”.

### 5.5 Summary of implementation challenges

Now that the ICH S1B(R1) Addendum (2022) has been adopted, it is important that industry and DRA scientists continue to communicate and collaborate to make the implementation of this addendum successful. The S1B(R1) IWG being established by ICH should provide the right forum for industry and DRAs to have this dialog. A close partnership between DRA and industry will ultimately result in reducing animal use, in accordance with 3R’s principles and an objective of the ICH S1B (R1) Addendum (2022), and optimizing resources for both Industry and DRAs without compromising the safety of medicines. It will be important, however, that industry submits only those WoE assessment with high confidence that a 2-year carcinogenicity study would not add value (either because of a high or a low carcinogenic risk); otherwise, this will end up increasing the DRA’s workload rather than decreasing it.

## 6 Case examples

Case examples are useful tools to illustrate how WoE factors are integrated to reach a decision on the appropriate carcinogenicity approach for a particular program. The ICH S1B(R1) Addendum (2022) includes an appendix which summarizes key attributes of 4 of the cases that were submitted in the PES. In addition, the paper of Bourcier et al. (2024) describing the results of the PES provides a tabulation of key features of each of the cases submitted to the PES. These examples are instructive for both sponsors and regulators in understanding key attributes that are important in determining the appropriate carcinogenicity assessment strategy.

To further supplement available case material, industry colleagues from the JPMA retrospectively reviewed publicly available data from marketed pharmaceuticals. For this exercise the presence or absence of one of the standard WoE factors was determined and an assessment was conducted to determine if the carcinogenic potential in humans was considered likely or unlikely. The general pharmacologic class of drug, summary of the WoE assessment, and rodent tumor outcomes were summarized in a series of tables and text that are provided in the Supplementary Material.

It is important to note that the case examples presented in the ICH S1B(R1) Addendum (2022), the Supplementary Material in this commentary, or in other forms are by necessity very high-level summaries of key illustrative concepts. As described in the addendum and this commentary, the documentation of a WoE assessments requires a scientifically robust and detailed analysis of the program that goes well beyond the key points capture in case summaries.

## 7 Conclusion

While the value of rodent carcinogenicity studies for safety assessment in the different sectors (pharmaceuticals, chemicals, foods) will continue to be debated for years to come, the ICH S1B(R1) Addendum (2022) provides the first notable change in the paradigm for pharmaceuticals since the development and implementation of medium-term mouse models in the mid-to-late 1990s. The addendum first directs sponsors to carefully consider all elements of a program to develop a carcinogenicity assessment strategy rather than adopting a check-the-box mentality that relies solely on rodent carcinogenicity studies to assess potential risk. To aid sponsors in this process, Section 2 of this paper reviewed key WoE factors and suggested approaches for sponsors to consider in conducting their assessments and deciding if the WoE is appropriate for their program. An important component of the addendum is the acknowledgement that investigative approaches can aid in carcinogenicity risk assessment by helping explain relevance of findings of concern observed in *in vivo* studies. To allow for sufficient flexibility and evolution of science the addendum was relatively brief and conceptual on this point, so Section 3 of this paper provides an expanded discussion of views from industry members of the EWG on the current status of these approaches. As this field of enquiry evolves it is our expectation that emerging technologies can be incorporated into the WoE paradigm on a more frequent basis without need for guidance revision. As outlined in the addendum, regulatory input is required before proceeding with registration. As highlighted in Section 4 sponsors must ensure that their WoE assessments are rigorous and carefully documented and presented to regulators in a coherent manner. In addition to the scientific considerations described in this paper, there are several implementation challenges. Section 5 reviews some of these challenges that sponsors need to carefully consider in developing a carcinogenicity strategy. Finally, based on an initiative from our colleagues in JPMA, the paper has provided additional case examples based on a retrospective review of marketed pharmaceuticals. These cases may be useful for sponsors and regulators as they consider how to apply the WoE factors.

ICH S1B(R1) (2022) provides an opportunity to move drug development and regulatory review to a more mechanistic and hypothesis-driven approach to carcinogenicity assessment that would inform both sponsor and regulatory decision-making. This shift in assessment strategies would encourage a more proactive mindset, create meaningful dialog with regulatory scientists and minimize drug development delays or discontinuations relating to carcinogenic risk.

The arc of this most recent revision to pharmaceutical carcinogenicity assessments had its origins in data gathered by industry scientists over a decade ago, evolved through multiple analyses by consortia and DRAs, and ultimately a prospective data collection and analysis that enabled the revision. Despite the substantial work and progress to date, much work remains for sponsors to effectively implement WoE approaches by conducting rigorous scientific reviews, implementing when appropriate investigational approaches, and finally presenting regulators with clear and complete dossiers to support the assessment. For DRAs, much work also remains in terms of providing consistent and timely reviews and seeking opportunities to share experiences and learning across regions so there is even greater global harmonization. Ultimately these efforts should result in a more rigorous and thoughtful approach to carcinogenicity testing that decreases animal use without compromising patient safety.

## Data Availability

The original contributions presented in the study are included in the article/Supplementary Material, further inquiries can be directed to the corresponding author.

## Footnotes

1 OECD https://www.oecd.org/chemicals

2 AOP WIKI https://aopwiki.org/
