# Supplementary material for: ICH S1 prospective evaluation study and weight of evidence assessments: commentary from industry representatives
Source: Front Toxicol. 2024 May 23;6:1377990. doi: 10.3389/ftox.2024.1377990 (PMC11153695; doi:10.3389/ftox.2024.1377990)
Supplement: Supplementary file 1 [file DataSheet1.PDF]

Supplemental Information

Case Examples Derived From Marketed Pharmaceuticals

Examples for when the carcinogenic potential in humans is considered LIKELY

When the target biology of the drug results in broad and potent immunosuppression, a potential human risk exists. Also, some agents that have a hormonal mechanism may present a well-known human risk. In these cases, if there are no off-target effects suggesting other carcinogenicity risk factors, it may be appropriate for the sponsor to conclude that the drug is LIKELY to be carcinogenic in humans and to conclude that a 2-year rat (2YR) study would not add value to the human risk assessments.

The indication of Drug-1 and Drug-2 is prostate cancer. The actual outcome of the 2YR study for Drug-1 was only an increase in Leydig cell tumors, which was considered a rodent-specific tumor. On the other hand, Drug-2 induced an increase in tumors of the pituitary, thymus, mammary glands, ovary, testis, and urinary bladder. If we do not conduct a 2YR study, these drug labels are likely to be similar and stating that carcinogenic potential in hormone sensitive organs cannot be excluded in humans. In the case of Drug 1, the result from the 2YR study provides the sponsor the accurate carcinogenic profile in rats and exposure multiples to the carcinogenic dose. It may benefit the sponsor if the sufficient exposure multiple is demonstrated. If not, even Leydig cell tumor may be interpreted as a flag that suggests potential tumorigenicity based on its hormonal action although it is not a direct risk in humans. In the case of Drug 2, a Weight of Evidence (WoE) approach benefited the sponsor in terms of time, budget and animal use perspectives rather than to perform a 2YR study.

As it is stated in S1B(R1), evidence of broad immunosuppression may provide sufficient concern for human risk that would not be further informed by standard rat and mouse carcinogenicity studies. The value of 2YR study for immunosuppressants is lower than other LIKELY cases although tumor outcomes vary among Drug 7-10. When findings of carcinogenic concern (FCC) outside immunosuppression are detected as off target effects or general toxicity studies, a 2-year rat study may be informative for evaluating carcinogenicity by off-target mode of action.

Supplemental Table A: Marketed drugs which could be judged as carcinogenic risk in humans are LIKELY by WoE approach

Product	Profile	WoE factors						Tumor outcome
		1	2	3	4	5	6	
Drug 1	Androgen synthesis inhibition	X	X	X	X			TgM: ---; 2YM: no data; 2YR: Leydig cell tumor

Drug 2	Antiandrogen	X	X	X	X			TgM: ---; 2YM: no data; 2YR: Leydig cell tumor, Pituitary, Thymus, M. Gland, Ovary, Urinary bladder
Drug 3	Estrogen	X			X			TgM: ---; 2YM: Mammary gland; 2YR: Pituitary, Mammary gland
Drug 4	SERM	X		X	X			TgM: Ovary; 2YM: no data; 2YR: Ovary, Kidney
Drug 5	SERM	X		X	X			TgM: ---; 2YM: Ovary, Leydig cell tumor, Prostate; 2YR: Ovary, Kidney
Drug 6	Immunosuppressor	X		X			X	TgM: Stomach, Skin; 2YM: Thymus; 2YR: ---
Drug 7	Immunosuppressor (JAK inhibition)	X		X			X	TgM: ---; 2YM: no data; 2YR: ---
Drug 8	Immunosuppressor (JAK inhibition)	X		X	X		X	TgM: ---; 2YM: no data; 2YR: Leydig cell tumor
Drug 9	Immunosuppressor (JAK inhibition)	X		X	X		X	TgM: ---; 2YM: no data; 2YR: Leydig cell tumor, Adipose tissue, Thymus, Lymph node
Drug 10	Immunomodulation	X		X			X	TgM: Hemangioma/sarcoma; 2YM: no data; 2YR: ---
Drug 11	PPAR gamma agonist	X		X				TgM: Hemangioma/sarcoma; 2YM: Hemangioma/sarcoma, Liver; 2YR: ---
Drug 12	Bcr-Abl tyrosine kinase inhibitor	X		X	X			TgM: Skin; 2YM: no data; 2YR: ---

X, Information/findings of carcinogenic concern exist

WoE 1, Drug target biology and the primary pharmacologic mechanism of the parent compound and major human metabolites including carcinogenicity information on class effects; WoE 2, Secondary pharmacology screens; WoE 3, Histopathology from 6-month rat study; WoE 4, Hormonal perturbation; WoE 5, Genetic toxicology study data; WoE 6, Immune modulation

Additional Details on Selected Cases

1.1 Drug-12

In addition to hormonal action and immune modulation, information on the drug class are important. The primary pharmacology of Drug-12 is inhibition of Bcr-Abl tyrosine kinase which is involved in abnormal proliferation in hematopoietic stem cells. A drug with the same pharmacology induced benign/malignant tumors in the kidney, stomach, intestine, adrenal perpetual/clitoral gland in the 2YR study, while atrophic changes and cellular decreases were observed in the lymph nodes and thymus in the 2-week rat (2WR) study. Given the limited information from a short-term general toxicity study and the multi-site tumorigenicity in the 2YR study, tumorigenicity of Drug-12 cannot be ruled out. It could be LIKELY case, but some DRA may judge that the WoE approach is not sufficient when targets of tumorigenicity are not fully clarified. In global drug developments, sponsors need to conduct a 2YR study even one DRA disagrees to WoE assessments. The actual outcomes of the carcinogenicity studies of Drug-12 were positive in the TgM study (papilloma/carcinoma in the skin) and negative in the 2YR study.

Supplemental Table B Drug-12 (Target indication, Leukemia)

WoE 1	• Multi-protein tyrosine kinase inhibitor, targeting Bcr-Abl fusion protein, c-kit and PDGFR α and β.<br>• Major metabolites are unchanged body<br>• A drug in the same class induced benign/malignant tumors in the stomach, intestine, adrenals, kidney, perpetual/clitoral gland in 2YR study when atrophic change and cellular decrease were observed in the lymph nodes and thymus in 2WR study
WoE 2	No effects on protein kinase other than inhibitory effects on PDGFR, c-Kit. (Uncertainty arise from the fact that only kinases were evaluated)
WoE 3	• 6MR study: Uterine dilation (60 mg/kg/day, 1.7X AM), although only hyaline droplets were shown in 1MR study<br>• 9MM study: Bile duct hyperplasia, Periductal fibrosis, (≥30 mg/kg/day, 0.3X AM), Sinusoidal cell hypertrophy / hyperplasia / cytoplasmic aggregation (≥200 mg/kg/day, 0.4X AM)
WoE 4	• 6MR study: Increased ovarian weight (≥6 mg/kg/day, 0.4X AM), uterine dilatation (≥ 60 mg/kg/day, 1.7X AM)<br>• Embryo and fetal mortality (≥20 mg/kg/day, 4.1X AM)
WoE 5	Negative
WoE 6	No evidence

AM, AUC margin to maximum recommended human dose; PDGFR, Platelet-derived growth factor receptor; 2WR study, 2-week rat study; 6MR study, 6-month rat study; 9MM study, 9-month monkey study
Underlined, Findings of carcinogenic concern
WoE 1, Drug target biology and the primary pharmacologic mechanism of the parent compound and major human metabolites including carcinogenicity information on class effects; WoE 2, Secondary pharmacology screens; WoE 3, Histopathology from 6-month rat study; WoE 4, Hormonal perturbation; WoE 5, Genetic toxicology study data; WoE 6, Immune modulation

Examples for when the carcinogenic potential in humans is considered UNLIKELY

During the prospective evaluation study, 31 UNLIKELY case CADs (Category 3a+3b) were submitted by PhRMA, EFPIA, and JPMA member pharmaceutical companies. However, only 12 cases were able to reach unanimous agreements by the DRAs. The rest of the cases fell into Category 2 (Uncertain carcinogenic potential), otherwise the decisions were split. In other words, at least one DRA considered a 2YR study to be of value in 19 cases. Therefore, the unanimous agreement to the UNLIKELY assessment is considered more difficult than to the LIKELY assessment. Comprehensive review and documentation are required, especially for FIC drugs.

Supplemental Table C Marketed drugs which could be judged as carcinogenic risk in humans are UNLIKELY by WoE approach

Product	Profile	WoE factors						Tumor outcome
		1	2	3	4	5	6	
Drug A	Orexin-1 and orexin-2 antagonist	X	X					TgM: ---; 2YR: Liver, Thyroid
Drug B	Viral RNA polymerase			X				TgM: ---; 2YR: ---

Drug C	DPP-4 inhibitor			X	X			TgM: ---; 2YR: ---
Drug D	Non absorbable phosphate binder				X			TgM: Ovary; 2YR: ---

X, Information/findings of carcinogenic concern exist
WoE 1, Drug target biology and the primary pharmacologic mechanism of the parent compound and major human metabolites including carcinogenicity information on class effects; WoE 2, Secondary pharmacology screens; WoE 3, Histopathology from 6-month rat study; WoE 4, Hormonal perturbation; WoE 5, Genetic toxicology study data; WoE 6, Immune modulation

Additional Details on Selected Cases

2.1 Drug-A

The indication for Drug A is insomnia. The pharmacological target is the orexin-1 and orexin-2 receptor, which regulate sleep and appetite, and are not thought to be involved in cellular proliferation. In WoE 1, orexin is known to be involved in luteinizing hormone secretion. Carcinogenicity studies of drugs in the same class were negative. In WoE 2, off-target binding to dopamine, which is related to prolactin secretion, was demonstrated. However, no related findings were detected in WoEs 3 and 4. The gastro-intestinal toxicity in the 6-month rat (6MR) study was observed where AUC margin was 43X or higher and was attributed to local irritation of dosing formulation. The AUC margin relative to the NOAEL is sufficient to indicate that the risk to humans was reduced. Taken together, a WoE assessment may have encouraged the sponsor to conclude that the carcinogenic risk of Drug A is less likely in humans. Actual outcome results were negative in TgM and tumors of low human relevance in 2YR (hepatocellular adenoma in liver and thyroid follicular cell adenoma).

Supplemental Table D Drug-A (Target indication, insomnia)

WoE 1	• Selective orexin-1 and orexin-2 receptor antagonist<br>• Orexin stimulates food intake. Modulates pituitary luteinizing hormone secretion in an ovarian steroid-dependent manner.<br>• A drug in the same class, Drug-A1, did not induce tumor in carcinogenicity studies.<br>• No active metabolite
WoE 2	• 165 GPCRs, transporters, ion channels, and enzymes<br>• Parent: Dopamine transporter at 3.8 μM, A3 receptor at 3.9 μM, Monoamine transporter at 4.2 μM, Na channel at 7 μM, hERG at 5.5 μM<br>• Metabolite X: Dopamine transporter at 10 μM
WoE 3	• 1MR study: Enzyme induction related changes in 1MR study (3MR study was skipped)<br>• 6MR study: Rodent-specific hepatic enzyme induction was suggested by hepatocellular hypertrophy and follicular cell hypertrophy in thyroid gland (≧19-23X AM); Mucosal necrosis in duodenum and glandular mucosal erosion in stomach (43-76X AM), NOAEL: 19-23X AM<br>• 9MD study (NOAEL: 42-48X AM): No histopathological finding (up to 77-92X AM)
WoE 4	• Nature of orexin that regulates LH<br>• Increases in TSH<br>• No evidence up to 43-76X AM in EFD; up to 86X AM in DART; up to 43-76X AM in chronic TOX
WoE 5	Negative

WoE 6	No evidence

AM, AUC margin to maximum recommended human dose; GPCR, G-protein coupled receptor; 1MR study, 1-month rat study; 6MR study, 6-month rat study; 9MD study, 9-month dog study

Underlined, Findings of carcinogenic concern

WoE 1, Drug target biology and the primary pharmacologic mechanism of the parent compound and major human metabolites including carcinogenicity information on class effects; WoE 2, Secondary pharmacology screens; WoE 3, Histopathology from 6-month rat study; WoE 4, Hormonal perturbation; WoE 5, Genetic toxicology study data; WoE 6, Immune modulation

2.2 Drug-B

The indication for Drug-B is antiviral (hepatitis C virus). The pharmacological target is viral origin RNA polymerase. An active metabolite has been identified and fully evaluated with the parent compound in general toxicity studies. Drug-B is considered FIC as a non-nucleic acid analogue type NS5B inhibitor. Nucleic acid analog type Drug-B1, which shares the same pharmacological target, was negative in 2YM and 2YR studies. Binding to PDE4 is not considered a finding of carcinogenic concern (FCC). In the 6MR study, gastrointestinal findings including hyperplasia were observed but it was only at the highest dose where AUC margin was 79X. Although there was a slight decrease in lymphocytes or white blood cells (WBC) in the 6MR study, no histopathologic changes were observed at doses up to 79X the clinical exposure. No evidence of immunomodulation in dogs or humans. Taken together, a WoE assessment may have encouraged the sponsor to conclude that the carcinogenic risk in humans was an unlikely case. Actual outcomes were negative in TgM and hepatocellular adenoma in 2YR, which is generally interpreted as having low human relevance.

Supplemental Table E Drug-B (Target indication, antiviral)

WoE 1	• A viral NS5B (RNA polymerase) inhibitor (Non-nucleic acid analogue).<br>• Main human metabolites: M1 (25% of the parent exposure), which has pharmacological activity similar to the parent<br>• Drug-B1 (NS5B inhibitor, nucleic acid analogue type) was negative in 2YM and 2YR studies
WoE 2	• >50% binding to PDE4 (Parent and M1) @6.6 and 6.5 µg/mL<br>• Parent: PDE4 IC50, 1.3 µg/mL
WoE 3	• 1MR study (3MR study was skipped): No FCC<br>• 6MR study (5, 20, 80 mg/kg/day, NOAEL: Parent, 10X AM; M1, 0.43X AM): Increased liver weight and hypertrophy of hepatocytes (≥5 mg/kg/day: Parent, 1.2X AM; M1, 0.07X AM); Crypt epithelial apoptosis and <u>hyperplasia in the small intestine</u>; crypt epithelial apoptosis accompanied with mixed cellular infiltration at the base of the gastric mucosa (80 mg/kg/day: Parent, 79X AM; M1, 16X AM)<br>• 9MD study (1, 5, 25 mg/kg/day, NOAEL: Parent, 65X AM; M1, 63X AM): No adverse finding up to 25 mg/kg/day
WoE 4	No evidence

WoE 5	Negative
WoE 6	• Rat: Slight decrease of lymphocyte or WBC in 6MR study (Parent,79X AM; M1, 16X AM). Slight single cell necrosis in the GALT and mesenteric lymph node in 2WR Tox (Parent,10X AM; M1, 0.20X AM)<br>• Dog, Human: No evidence

AM, AUC margin to maximum recommended human dose; GALT, gut-associated lymphoid tissue; WBC, white blood cells; 2WR study, 2-week rat study; 6MR study, 6-month rat study; 9MD study, 9-month dog study

Underlined, Findings of carcinogenic concern

WoE 1, Drug target biology and the primary pharmacologic mechanism of the parent compound and major human metabolites including carcinogenicity information on class effects; WoE 2, Secondary pharmacology screens; WoE 3, Histopathology from 6-month rat study; WoE 4, Hormonal perturbation; WoE 5, Genetic toxicology study data; WoE 6, Immune modulation

2.3 Drug-C

DPP-4 inhibition is a common pharmacological action for the treatment of type II diabetes. Five drugs of the same class (Drug-C1, C2, C3, C4 and C5) were identified. Although some of the carcinogenicity studies were positive, tumor types and species were not consistent suggesting that these tumors may not be related to DPP4 inhibition but rather to other attributes of the compounds. This scenario might suggest the need for a 2-year study rather than relying on prior data from the class. Although hormonal actions were not expected based on WoEs 1 and 2, proliferative, atrophic, degenerative findings in hormone sensitive organs were seen in the 3MR and 6MR studies. However, AUC margins were more than 100X in both studies. In addition, findings in hormone sensitive organs reduced in the 6MR study. Measurement of hormone levels in cynomolgus monkeys or clinical trial samples may be useful to strengthen the interpretation. Necrotizing cutaneous lesions in cynomolgus monkeys are considered a class effect of DPP4 inhibitors. In general, chronic inflammation is considered FCC, but sufficient safety margin is achieved. DPP-4 is one of the T cell surface antigens, but no effect on T cell proliferation or mixed lymphocyte reaction was observed. Changes in WBC were slight. Overall, WoE assessment may have suggested that carcinogenic risk in human was a UNLIKELY case, but it may have been controversial at the end of the 3MR study. Actual outcomes were negative in TgM and in 2YR.

Supplemental Table F Drug-C (Target indication, Type II diabetes)

WoE 1	• DPP-4 inhibitor, pharmacological activity of major human metabolite (M1) is 1/39 of the parent compound<br>• Other DPP-4 inhibitors include Drug-C1 (hepatocellular adenoma/carcinoma in mice, negative in rats); Drug-C2 (mammary carcinoma and hemangiosarcoma in mice, negative in rats); Drug-C3 (negative in mice or rats); Drug-C4 (negative in mice or rats); Drug-C5 (hemangiosarcoma in rats, negative in mice)
WoE 2	• Parent, Inhibitory effects on DPP8/9, FAP, Histamine H1R and Sigma 1R<br>• M1, Inhibitory effects on DPP8/9, FAP, Histamine H1R
WoE 3	• 3MR study: Adrenal zona glomerulosa thickening / zona reticularis vacuolation; testicular retention,

	degeneration, atrophy; epididymis, increased degenerated cells; prostate and seminal vesicle, decreased choroid at 102-108X AM<br>•6MR study: NOAEL at 3-4X, Increased weight and aggregation of alveolar macrophage in lung; skin thickening; reduced weight and decreased choroid in seminal vesicle; testicular retention at 128-129X AM<br>•12MM study: NOAEL at 14-24X AM, necrotizing cutaneous lesions at 44-46X AM
WoE 4	Findings in male genital organs (3MR study > 6MR study)
WoE 5	Negative
WoE 6	•6MR study: elevated WBC (Neutrophil; basophil; Lymphocyte; monocyte, large unstained cell) counts at 128-129X AM<br>•Immuno-tox: No effects on T cell proliferation or Mixed lymphocyte reaction assays<br>•1MR study immuno-tox: Slight changes that are not immuno-toxicity concern (peripheral blood, CD45RA+ B cell count↑, CD3+ T cell count↑, CD26+ B cell count↓; Spleen, CD45RA+ B cell % ↑, CD3+ T cell %↓)

AM, AUC margin to maximum recommended human dose; FAP, fluorogen-activating protein; 1MR study, 1-month rat study; 6MR study, 6-month rat study; 12MM study, 12-month monkey study

Underlined, Findings of carcinogenic concern

WoE 1, Drug target biology and the primary pharmacologic mechanism of the parent compound and major human metabolites including carcinogenicity information on class effects; WoE 2, Secondary pharmacology screens; WoE 3, Histopathology from 6-month rat study; WoE 4, Hormonal perturbation; WoE 5, Genetic toxicology study data; WoE 6, Immune modulation

2.2 Drug-D

The indication for Drug-D is anti-hyperphosphatemia. This drug is considered FIC based on its attribute of being a non-absorbable type of phosphate binder. It is stable in gastric or intestinal juices. Metabolites and degradants have not been detected in the peripheral blood. Although some other absorbable phosphate binders were positive in carcinogenicity studies in mice or rats, the tumor types and species were not consistent like the Drug-C case. In the chronic toxicity study in rats and dogs, there were no remarkable histopathological changes, but the dose ratios (DR) to maximum recommended human dose were 16X DR and 5.3X DR, respectively. In the embryo-fetal development study in rats and rabbits, delayed/incomplete ossification was observed. Fetal and maternal mortality were also observed. The sponsor described that there were no changes in parathyroid hormone levels in the 1-month rat (1MR) study; blood calcium levels and histopathology in bones and parathyroids in the 6MR study. Delayed/incomplete ossification may be due to reduced Ca levels following phosphate binding, but this was not fully evaluated. Overall, given that Drug-D is not absorbable, DRA may support WoE approach rather than to perform 2YR study. However, when pharmacology exists, toxicology is expected, too. It may not be enough only to say that the drug is not absorbable. Comprehensive consideration is required to explain changes in embryo-fetal development studies in the briefing document.

Supplemental Table G Drug-D (Anti-hyperphosphatemia)

WoE 1	• Phosphate binder (FIC for non-absorbable drug)<br>• No absorbable metabolite/degradant (Stable in artificial gastric or intestinal juices)<br>• Phosphate toxicity can promote tumorigenesis (De-risk by normalized phosphate level)<br>• Other phosphate binders include Drug-D1 (adenocarcinomas in the colon in mice / negative in rats); Drug-D2 (urinary bladder transitional cell papilloma in rats / negative in mice); Drug-D3 (negative in mice or rats); Drug-D4 (glandular stomach adenomas in mice / negative in rats)
WoE 2	No secondary pharmacology screen (Not issue due to no absorption of Drug-D and no metabolite in gastrointestinal tract)
WoE 3	• 6MR study: No remarkable histopathologic change up to 16X DR<br>• 9MD study: No remarkable histopathologic change up to 5.3X DR
WoE 4	• 1MR study: No evidence up to 16X DR (including no changes in parathyroid hormone levels)<br>• 6MR study: No evidence up to 16X DR (including. no changes in blood Ca levels, or histopathology in bones or parathyroids)<br>• 9MD study: No evidence up to 5.3X DR<br>• Rat EFD: no evidence up to 8.0X DR, ossification retarded/uncompleted at 16X DR<br>• Rabbit EFD: No evidence at 2.7X DR, increased post-implantation mortality and no/incomplete ossification ≥ 8.0X DR, decreased live fetus at 16X DR<br>• Rat PPND: No evidence up to 8.0X DR, decreased body weight gain before wean in F1 at 16X DR
WoE 5	Negative
WoE 6	No evidence up to 5.3X DR in dogs or 16X DR in rats

DR, Dose ratio to maximum recommended human dose; 1MR study, 1-month rat study; 6MR study, 6-month rat study; 9MD study, 9-month dog study

Underlined, Findings of carcinogenic concern

WoE 1, Drug target biology and the primary pharmacologic mechanism of the parent compound and major human metabolites including carcinogenicity information on class effects; WoE 2, Secondary pharmacology screens; WoE 3, Histopathology from 6-month rat study; WoE 4, Hormonal perturbation; WoE 5, Genetic toxicology study data; WoE 6, Immune modulation
